# HER2-mutated lung squamous cell carcinoma responding to trastuzumab deruxtecan followed by pyrotinib: a Case Report

**DOI:** 10.3389/fphar.2026.1862847

**Published:** 2026-06-18

**Authors:** Lou Liu, Jing-wen Zhang, Yi Fu, Jie Chen, Ju-ying Zhou, Chen-ying Ma

**Affiliations:** 1 Department of Radiation Oncology, The First Affiliated Hospital of Soochow University, Suzhou, China; 2 Department of Radiation Oncology, The Fourth Affiliated Hospital of Soochow University, Suzhou, China; 3 Department of Radiation Oncology, City of Hope National Medical Center, Duarte, CA, United States

**Keywords:** case report, HER2 exon 20 mutation, lung squamous cell carcinoma, pyrotinib, trastuzumab deruxtecan

## Abstract

**Introduction:**

HER2 (ERBB2) alterations are recognized oncogenic drivers in non-small cell lung cancer, but they are uncommon and poorly characterized in lung squamous cell carcinoma (LSCC).

**Case Presentation:**

We report a patient with initially resected stage IA2 LSCC who subsequently developed metastatic pulmonary recurrence. Molecular profiling of the resected tumor revealed an ERBB2 exon 20 p. G776V (p.Gly776Val) mutation, MET amplification, and copy-number deletions involving CDKN2A/B, MTAP, and TERT. Postoperative systemic therapy was administered as an individualized off-guideline strategy after multidisciplinary discussion. Following pulmonary recurrence and subsequent progression on platinum-based therapy, trastuzumab deruxtecan (T-DXd) achieved clinically meaningful disease control but was discontinued because of suspected drug-associated interstitial lung disease/pneumonitis complicated by pulmonary infection. Pyrotinib was subsequently administered and achieved disease control despite gastrointestinal intolerance. After further disease progression and clinical improvement of the prior pulmonary event, reduced-dose T-DXd was cautiously reintroduced, resulting in an additional radiologic response and approximately 7 months of disease control. The later clinical course was complicated by suspected treatment-associated cardiac dysfunction and terminal respiratory deterioration related to progressive pulmonary disease and infection.

**Conclusion:**

This case suggests that ERBB2 activating mutations may represent potentially actionable alterations in selected patients with recurrent LSCC. Comprehensive genomic profiling may identify rare therapeutic opportunities in this histologic subtype. However, individualized off-guideline treatment, HER2-directed therapy sequencing, and T-DXd rechallenge after pulmonary toxicity require cautious multidisciplinary risk–benefit assessment.

## Introduction

Lung cancer remains the leading cause of cancer-related mortality worldwide, with non–small cell lung cancer (NSCLC) accounting for 80%–85% of cases (Bray et al., 2024). Lung squamous cell carcinoma (LSCC), a major NSCLC subtype, is characterized by aggressive behavior and poor prognosis. Advances in genomic profiling have identified actionable oncogenic drivers, including human epidermal growth factor receptor 2 (HER2, ERBB2). HER2 alterations—most commonly exon 20 insertions—occur in 2%–4% of NSCLC (Tu et al., 2023), predominantly in adenocarcinoma, while remaining rare and poorly characterized in LSCC. Although HER2-targeted therapies, such as tyrosine kinase inhibitors and antibody–drug conjugates, have shown clinical activity in HER2-mutant NSCLC, evidence in LSCC is limited. Here, we describe a patient with recurrent LSCC harboring an ERBB2 exon 20 p. G776V (p.Gly776Val) mutation who achieved disease control with individualized HER2-directed therapy. This case highlights the potential value of comprehensive genomic profiling for identifying rare, potentially actionable alterations in squamous histologic contexts.

## Case presentation

### Patient information and initial presentation

A 69-year-old man was admitted in August 2022 after bilateral pulmonary nodules were incidentally detected during routine examination 1 month earlier. He reported an occasional productive cough but denied chest pain, dyspnea, hemoptysis, fever, or weight loss. His medical history was unremarkable, with no prior malignancy, thoracic surgery, smoking or alcohol use, and no family history of cancer. On admission, his Eastern Cooperative Oncology Group (ECOG) performance status was 0. Physical examination and vital signs were unremarkable.

### Diagnostic assessment

Chest computed tomography (CT) performed in August 2022 revealed a 19-mm nodule in the dorsal segment of the left lower lobe with shallow lobulation and a small internal cavity. The lesion showed marked contrast enhancement. On 15 September 2022, the patient underwent uniportal video-assisted thoracoscopic left lower lobectomy with regional lymph node dissection. Intraoperatively, a firm lesion measuring approximately 2.0 cm was identified in the dorsal segment of the left lower lobe, with focal pleural retraction but no pleural effusion.

The resected specimen was processed using standard surgical pathology procedures, including formalin fixation, paraffin embedding, sectioning, and haematoxylin and eosin staining. Histopathological examination confirmed poorly differentiated squamous cell carcinoma without pleural involvement. The bronchial margin was negative. Fourteen regional lymph nodes were negative for metastasis, including one peribronchial lymph node and 13 lymph nodes sampled from stations 5, 7, 9, 10 and 11.

Immunohistochemistry was performed on formalin-fixed, paraffin-embedded tumour sections using a standardized automated staining workflow with heat-induced epitope retrieval and DAB-based visualization. The diagnostic panel included markers of squamous differentiation, adenocarcinoma lineage, neuroendocrine differentiation, proliferation, MET expression and pleural elastic fibre involvement. Details of antibody clones, manufacturers, staining platform, antigen-retrieval conditions, detection systems, and positive and negative control procedures are provided in the Supplementary Protocol, Supplementary Table S1.

The tumour was positive for CK7, CK5/6, p40 and p63, and negative for TTF-1, Napsin A, ALK (D5F3), chromogranin A, synaptophysin and CD56, supporting squamous differentiation and excluding adenocarcinoma or neuroendocrine differentiation. The Ki-67 proliferation index was approximately 50%. MET immunostaining was weakly positive (1+), and elastic fibre staining showed no evidence of pleural invasion. Ki-67 was assessed as the proportion of viable tumour-cell nuclei showing definite immunoreactivity in representative high-proliferation areas, while excluding necrotic, crushed, haemorrhagic or poorly preserved regions. Final interpretation was made by experienced pathologists on the basis of morphology, immunophenotype, and internal and external quality controls.

Comprehensive molecular profiling was performed as part of routine clinical care by Geno-Truth Dx using a large-panel targeted next-generation sequencing assay. According to the confirmed testing source and report framework, the assay was based on target-region probe capture followed by next-generation sequencing, with a workflow that included nucleic acid extraction, library preparation, high-throughput sequencing, bioinformatic analysis, and clinical interpretation. Both tumor tissue and plasma circulating tumor DNA (ctDNA) were tested. The reporting framework used GRCh37/hg19 as the reference genome. Quality-control procedures included assessment of tumor cell content for tissue samples, DNA quantity and quality, base quality, sequencing depth, and target-region coverage.

Tissue-based testing of the resected lung tumor on 16 September 2022 identified an ERBB2 exon 20 p. G776V missense mutation, MET copy-number amplification at 7q31.2, and copy-number deletions involving CDKN2A, MTAP, CDKN2B, and TERT. Tissue tumor mutational burden (TMB) was 11.96 mutations/Mb, and microsatellite stability was confirmed. Plasma ctDNA testing on 19 October 2022 detected LRP1B p. R1464 and SMO p. L408V, with a plasma TMB of 3.99 mutations/Mb and microsatellite stability. A subsequent tissue-based assay performed on 4 November 2022 again detected ERBB2 p. G776V, with a tissue TMB of 6.98 mutations/Mb and microsatellite stability. Programmed death-ligand 1 expression was low, with a tumor proportion score of 1% and a combined positive score of 1%. The tissue- and plasma-based genomic findings are summarized in Table 1.

**TABLE 1:** Tissue and plasma genomic profiling findings.

Date	Sample	Testing source/Assay	Key findings	TMB/MSI	Comment
2022-09-16	Lung tumor tissue	Geno-truth dx; large-panel targeted NGS	ERBB2 exon 20 p.G776V; MET copy-number amplification at 7q31.2; CDKN2A, MTAP, CDKN2B, TERT copy-number deletions	TMB 11.96 mut/Mb; MSS	VAF, absolute copy-number value, sample-specific depth, and MET amplification threshold were not available in the retrospective record
2022-10-19	Plasma ctDNA	Geno-truth dx; plasma ctDNA NGS	LRP1B p.R1464; SMO p.L408V	TMB 3.99 mut/Mb; MSS	ERBB2 p.G776V and MET amplification were not listed in the available plasma summary; plasma detectability could not be established from the available record
2022-11-04	Lung tumor tissue	Geno-truth dx; tissue NGS	ERBB2 p.G776V	TMB 6.98 mut/Mb; MSS	Repeat tissue testing confirmed ERBB2 p.G776V; transcript-level and cDNA-level HGVS notation was not available

ctDNA, circulating tumor DNA; MSI, microsatellite instability; MSS, microsatellite stable; NGS, next-generation sequencing; TMB, tumor mutational burden; VAF, variant allele frequency. The exact panel size, full gene list, patient-specific sequencing depth, VAFs, absolute copy number values, bioinformatic pipeline, and assay-specific threshold for defining *MET* amplification were not available in the retrospective record.

Although the testing source and general assay framework were confirmed, the original retrospective report did not provide the exact panel size, full gene list, patient-specific sequencing depth, variant allele frequencies, absolute copy-number values, bioinformatic software pipeline, or assay-specific threshold used to define MET amplification. The final diagnosis was poorly differentiated LSCC, stage IA2 (pT1bN0M0, AJCC 8th edition), with an ERBB2 exon 20 mutation and multiple co-occurring genomic alterations.

### Therapeutic interventions

Although the patient had completely resected stage IA2 LSCC, postoperative systemic therapy was not guideline-standard for this stage. After multidisciplinary discussion, postoperative chemoimmunotherapy was administered as an individualized off-guideline strategy, considering the poorly differentiated histology and multiple potentially high-risk molecular alterations, including an ERBB2 activating mutation, MET amplification, and CDKN2A/B-MTAP-TERT copy-number deletions. The limited evidence supporting this approach was discussed with the patient, and informed consent was obtained. The complete systemic treatment course is summarized in Table 2.

**TABLE 2:** Summary of systemic therapies, responses, progression-free survival, and key safety events.

Line	Clinical setting	Regimen	Dose/Schedule in available record	Period	Best response/Outcome	Clinical notes
1st	Postoperative individualized therapy	Albumin-bound paclitaxel + carboplatin + serplulimab; later serplulimab maintenance; one cycle serplulimab + anlotinib	Paclitaxel albumin 400 mg D1; carboplatin 0.5 g D1; serplulimab 300 mg D1; anlotinib 12 mg D1-14 for one cycle	November 2022-September 2023	Radiographically disease-free until pulmonary recurrence; PFS approx. 13 months	No measurable postoperative disease was present; this interval is described as radiographically disease-free surveillance rather than RECIST CR. Postoperative systemic therapy was individualized and off-guideline
2nd	Metastatic pulmonary recurrence	Gemcitabine + nedaplatin + anlotinib	Gemcitabine 1.4 g D1/8; nedaplatin 110 mg D2; anlotinib 12 mg D1-14	September-November 2023	PD; PFS approx. 2 months	Pulmonary recurrence was diagnosed radiologically; re-biopsy was not documented
3rd	ERBB2-mutant recurrent LSCC	T-DXd	Fixed dose 300 mg Q3W; 4 cycles	November 2023-April 2024	Disease control; later PD; PFS approx. 5 months	The available record documented a fixed dose; body weight-based dose calculation was not available
Safety event after 3rd-line therapy	T-DXd interruption	Nintedanib + corticosteroid + anti-infective management	Nintedanib 100 mg twice daily; methylprednisolone dose/taper unavailable	February-March 2024	Pulmonary findings improved after treatment	Pulmonary event was managed as suspected T-DXd-associated ILD/pneumonitis complicated by documented pulmonary infection
4th	After T-DXd interruption	Pyrotinib	320 mg QD reduced to 240 mg; intermittent dosing due to GI intolerance	April-August 2024	Disease control/transient radiologic improvement; PFS approx. 4 months	Response is conservatively described as disease control/transient radiologic improvement because complete RECIST measurements were unavailable and infection absorption may have contributed
5th	After pyrotinib progression	Cadonilimab + anlotinib	Cadonilimab + anlotinib; Cadonilimab 500 mg; anlotinib 8 mg; docetaxel not confirmed in current history	August-September 2024	PD; PFS approximately 1 month	Docetaxel was not included because its administration was not confirmed in the available record
6th	T-DXd rechallenge	T-DXd	Reduced fixed dose 200 mg Q3W; 11 cycles in record	September 2024-April 2025	PR/disease control; PFS approx. 7 months	Reduced-dose rechallenge was undertaken cautiously after clinical improvement of the prior pulmonary event and risk discussion
7th	Terminal phase	Best supportive care	High-flow oxygen, anti-infective/antiviral/bronchodilator/corticosteroid/diuretic/supportive therapy	May 2025	Hospice discharge on 30 May 2025	Follow-up was calculated to hospice discharge on 30 May 2025 because a confirmed date of death was not available in the record

D, day; GI, gastrointestinal; ILD, interstitial lung disease; LSCC, lung squamous cell carcinoma; PD, progressive disease; PFS, progression-free survival; Q3W, every 3 weeks; RECIST, Response Evaluation Criteria in Solid Tumors; T-DXd, trastuzumab deruxtecan; QD, once daily.

From 25 November 2022 to 18 March 2023, the patient received six cycles of albumin-bound paclitaxel, carboplatin, and serplulimab. One cycle of serplulimab plus anlotinib was administered on 10 April 2023; however, anlotinib was discontinued because of epistaxis and fatigue. Serplulimab maintenance was continued until 14 September 2023. The patient remained radiographically disease-free during postoperative systemic therapy until pulmonary recurrence was detected in September 2023.

Surveillance CT on 13 September 2023 showed interval enlargement of multiple bilateral pulmonary nodules, consistent with pulmonary recurrence. The recurrence was diagnosed radiologically based on the multiplicity and interval growth of bilateral pulmonary nodules on serial CT imaging. Re-biopsy of the recurrent pulmonary nodules was not documented in the available medical record.

Given the ERBB2 exon 20 activating mutation, trastuzumab deruxtecan (T-DXd) was initiated on 13 November 2023 at the documented fixed dose of 300 mg every 3 weeks. Four cycles were administered until 16 January 2024. CT performed on 5 February 2024 showed progressive fibrosing interstitial lung abnormalities. T-DXd was discontinued, and nintedanib 100 mg twice daily was initiated.

The patient subsequently developed exertional dyspnea and productive cough. Repeat CT on 29 February 2024 showed progression of bilateral pulmonary inflammatory changes. He was treated with broad-spectrum antibiotics, methylprednisolone, mucolytic therapy, gastric protection, and renal protection. Microbiological work-up showed positive fungal markers, and sputum next-generation sequencing later detected *Klebsiella pneumoniae* and *Pneumocystis jirovecii*. The pulmonary event was therefore managed as suspected T-DXd-associated interstitial lung disease/pneumonitis complicated by superimposed pulmonary infection. His respiratory symptoms and radiologic findings improved after antifibrotic, anti-inflammatory, and anti-infective treatment, and long-term oral isavuconazole was continued after discharge. The exact corticosteroid dose, tapering schedule, oxygen requirement at onset, standardized high-resolution CT pattern, and formal Common Terminology Criteria for Adverse Events grade were not completely documented in the retrospective record.

After progression on the first T-DXd exposure, pyrotinib was initiated on 16 April 2024 at 320 mg once daily and was subsequently reduced to 240 mg because of nausea and vomiting. Imaging in May 2024 showed improvement of pulmonary inflammatory changes and reduction of left lower lung abnormalities, which was interpreted clinically as radiologic improvement. Because post-infectious absorption may have contributed to these imaging changes and treatment adherence was intermittent because of gastrointestinal intolerance, the best response to pyrotinib was conservatively described as disease control or transient radiologic improvement. CT on 20 August 2024 showed progression of bilateral pulmonary nodules, corresponding to an estimated progression-free survival of approximately 4 months.

After progression on subsequent therapy and clinical improvement of the prior pulmonary event, the case was re-evaluated. Given the previous clinical benefit from T-DXd, limited remaining treatment options, and patient and family preference after risk discussion, T-DXd rechallenge was undertaken at a reduced fixed dose of 200 mg every 3 weeks beginning on 12 September 2024. Serial CT imaging showed a partial response in November 2024 and persistent tumor control in February 2025. Disease progression was confirmed in April 2025. The estimated progression-free survival during T-DXd rechallenge was approximately 7 months. Body weight-based dose calculation was not available in the retrospective record.

During T-DXd rechallenge, the patient developed left anterior chest pain in January 2025. Twenty-four-hour Holter monitoring showed occasional atrial premature beats, frequent ventricular premature beats, and ST-T abnormalities. Echocardiography demonstrated basal interventricular septal thickening, mild mitral regurgitation, reduced left ventricular systolic function, and a small pericardial effusion. CT showed postoperative changes in the left lung and bilateral pulmonary inflammatory changes. Electrocardiography on 12 February 2025 showed sinus rhythm with first-degree atrioventricular block. The patient was managed by the cardiology team with anti-remodeling, inotropic, diuretic, and vasodilatory therapy. Because complete serial left ventricular ejection fraction values, cardiac biomarkers, and coronary evaluation were unavailable, the event was described as suspected treatment-associated cardiac dysfunction rather than definitively attributed to T-DXd.

### Follow-up and outcomes

**FIGURE 1:**
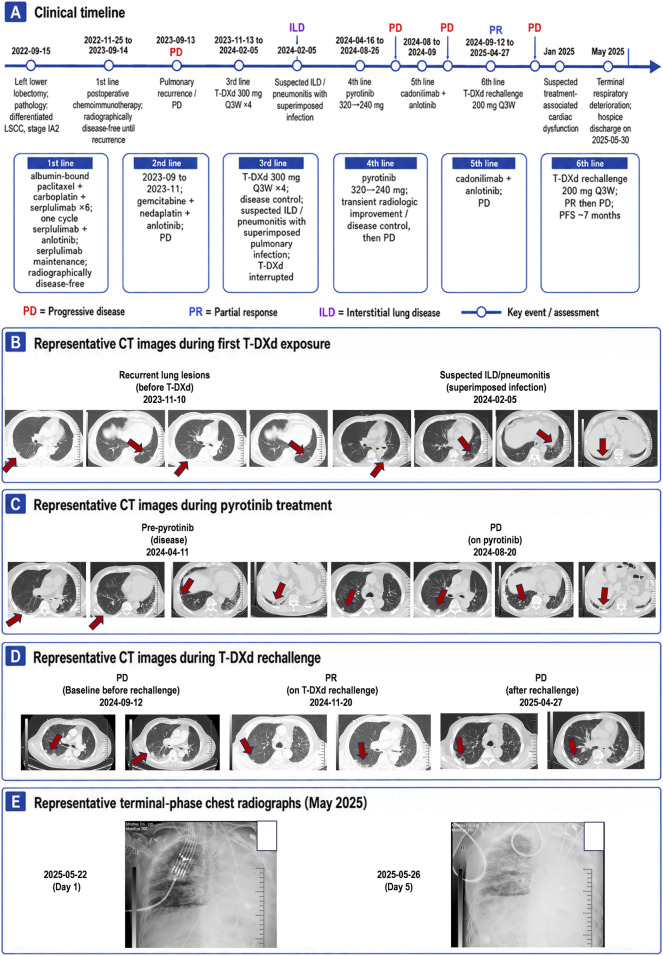
Clinical course and representative thoracic imaging. **(A)** Timeline of systemic therapies, radiologic assessments, and major safety events from initial diagnosis to hospice discharge. **(B)** Representative CT images during the first trastuzumab deruxtecan (T-DXd) exposure, showing radiologic tumor changes and subsequent pulmonary interstitial/inflammatory abnormalities. **(C)** Representative CT images during pyrotinib treatment. **(D)** Representative CT images during T-DXd rechallenge, showing radiologic response followed by disease progression. **(E)** Representative imaging during terminal respiratory deterioration. Arrows indicate representative target lesions, pulmonary interstitial/inflammatory abnormalities, or pleural effusion. Abbreviations: ADC, antibody–drug conjugate; ILD, interstitial lung disease; PD, progressive disease; PFS, progression-free survival; PR, partial response; SD, stable disease; T-DXd, trastuzumab deruxtecan; TKI, tyrosine kinase inhibitor.

**FIGURE 2:**
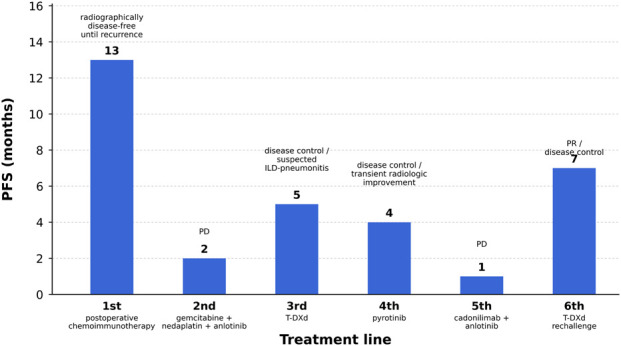
Progression-free survival across systemic treatment lines. Bars indicate progression-free survival (PFS) for each systemic treatment line, calculated from treatment initiation to radiologic progression or treatment discontinuation due to progression. Best response and key safety events are annotated. Overall follow-up duration is reported separately in the main text and is not plotted, to avoid conflating cumulative follow-up with line-specific PFS.

In May 2025, the patient was admitted with worsening chest tightness and dyspnea. He developed acute respiratory distress, severe dry cough, intermittent hemoptysis, tachycardia, tachypnea, and hypoxemia. Chest radiography showed postoperative changes in the left lung, pulmonary inflammation, multiple bilateral pulmonary nodules, a small right pleural effusion, and a large left pleural effusion. He received high-flow oxygen, broad-spectrum antibacterial therapy, antiviral therapy, bronchodilators, corticosteroids, diuretics, hemostatic therapy, and supportive care. His respiratory condition did not improve substantially, and the family declined invasive resuscitation. He was discharged for hospice care on 30 May 2025. The clinical timeline, representative thoracic imaging, treatment responses, and major safety events are shown in Figure 1. Line-specific progression-free survival is summarized in Figure 2. The follow-up duration from initial diagnosis to hospice discharge was approximately 33 months.

## Discussion

In the present case, an ERBB2 exon 20 p. G776V missense mutation was identified in lung squamous cell carcinoma (LSCC), a histologic subtype in which HER2 alterations are rarely reported and remain less well characterized than in lung adenocarcinoma. HER2 alterations occur in a small subset of non-small cell lung cancer (NSCLC), with most reported HER2-mutant cases arising in adenocarcinoma rather than squamous histology (Tu et al., 2023). This case raises the possibility that ERBB2 activating mutations may define a small subset of recurrent LSCC with potentially actionable disease. However, this observation should be interpreted as hypothesis-generating rather than practice-changing.

The patient harbored ERBB2 p. G776V together with MET copy-number amplification and copy-number deletions involving CDKN2A, MTAP, CDKN2B, and TERT, representing a complex genomic profile for an initially resected stage IA2 LSCC. Previous studies have associated HER2 alterations and MET amplification with adverse clinical features or poorer outcomes in NSCLC (Yin et al., 2021; Zhang et al., 2025). In this single case, these co-occurring alterations may suggest biologically aggressive disease, but a causal relationship with early postoperative recurrence cannot be established. The patient remained radiographically disease-free during postoperative surveillance but developed pulmonary recurrence within approximately 13 months. Although adjuvant immunotherapy has improved disease-free survival in selected patients with completely resected stage IB–IIIA NSCLC in KEYNOTE-091 (O'Brien et al., 2022), this evidence does not apply to stage IA2 disease. Therefore, postoperative systemic therapy in this case should be regarded as an individualized off-guideline decision made after multidisciplinary discussion, rather than as a model for routine management of completely resected stage IA NSCLC.

After metastatic pulmonary recurrence, HER2-directed therapy became the main biologically guided treatment strategy. The clinical activity of trastuzumab deruxtecan (T-DXd) in previously treated HER2-mutant NSCLC has been demonstrated in the DESTINY-Lung program, with objective responses and median progression-free survival supporting its role as an important HER2-targeted antibody–drug conjugate in this setting (Li et al., 2022; Cheng et al., 2024). In the present patient, T-DXd provided disease control during initial exposure and, after later rechallenge at a reduced dose, produced an additional radiologic response with approximately 7 months of disease control. The shorter initial benefit relative to trial medians may reflect the heavily pretreated setting, squamous histology, co-occurring MET amplification, and treatment interruption because of pulmonary toxicity. MET amplification is a recognized bypass mechanism in lung cancer and may contribute to resistance to targeted therapies, although this possibility cannot be confirmed in this case without longitudinal molecular monitoring (Qin et al., 2023).

Pyrotinib was associated with disease control or transient radiologic improvement after T-DXd interruption. However, early imaging improvement may have been confounded by resolution of pulmonary infection, and treatment adherence was intermittent because of gastrointestinal intolerance. Prior studies of pyrotinib in HER2-mutant NSCLC have reported antitumor activity, with response rates and median progression-free survival supporting its consideration as a later-line option where accessible; however, the evidence base remains less mature than that for T-DXd (Zhou et al., 2020; Song et al., 2022).

Interstitial lung disease (ILD)/pneumonitis is a major safety concern with T-DXd. In this patient, pulmonary interstitial and inflammatory abnormalities were detected during the first T-DXd exposure, but the clinical course was complicated by documented infection, including positive fungal markers and sputum next-generation sequencing detection of *Klebsiella pneumoniae* and *Pneumocystis jirovecii*. The pulmonary event is therefore best described as suspected T-DXd-associated ILD/pneumonitis complicated by superimposed pulmonary infection. Because the retrospective record did not fully document oxygen requirement at onset, standardized high-resolution CT pattern, corticosteroid dose and taper, or formal Common Terminology Criteria for Adverse Events grade, the event could not be graded reliably. Rechallenge after T-DXd-associated ILD/pneumonitis remains clinically sensitive. Emerging evidence suggests that retreatment after recovery from low-grade ILD may be feasible in carefully selected patients under close monitoring (Rugo et al., 2025; Yasuda et al., 2025), but such decisions require individualized risk–benefit assessment. In this case, reduced-dose T-DXd rechallenge was undertaken only after clinical improvement of the prior pulmonary event, in the context of limited remaining treatment options and after discussion with the patient and family.

The later clinical course was complicated by suspected treatment-associated cardiac dysfunction, including chest pain, Holter-detected atrial and ventricular premature beats, ST-T abnormalities, echocardiographic evidence of reduced left ventricular systolic function, and first-degree atrioventricular block. Because complete serial left ventricular ejection fraction values, cardiac biomarkers, and coronary evaluation were unavailable, definitive attribution to T-DXd cannot be made.

This case report has several limitations. First, it describes a single patient, and the therapeutic implications of HER2-directed therapy in LSCC remain uncertain. Second, pulmonary recurrence was diagnosed radiologically, and re-biopsy of recurrent lesions was not documented. Third, although the testing source and general Geno-Truth Dx large-panel targeted next-generation sequencing framework were confirmed, the exact panel size, full gene list, patient-specific sequencing depth, variant allele frequencies, absolute copy-number values, and MET amplification threshold were not available in the retrospective record. Fourth, the pulmonary toxicity during T-DXd treatment was complicated by documented pulmonary infection, making it difficult to distinguish drug-related ILD/pneumonitis from superimposed infectious pneumonia. Fifth, several treatments, including postoperative systemic therapy for stage IA2 disease and T-DXd rechallenge, were individualized off-guideline decisions made in a complex clinical context. Finally, complete serial RECIST target-lesion measurements and longitudinal laboratory safety data, including complete blood count, liver and renal function tests, inflammatory markers, cardiac biomarkers, and left ventricular ejection fraction measurements, were not available, limiting formal response assessment, comprehensive safety evaluation, and definitive attribution of the observed cardiac dysfunction to T-DXd.

Overall, this case supports comprehensive genomic profiling in recurrent LSCC and suggests that ERBB2 activating mutations may occasionally reveal therapeutic opportunities beyond adenocarcinoma. At the same time, off-guideline postoperative therapy, HER2-directed treatment sequencing, T-DXd rechallenge after pulmonary toxicity, and interpretation of benefit after multiple treatment lines require cautious multidisciplinary assessment.

## Established facts and novel insights

### Established facts

Lung cancer is the leading cause of cancer-related death worldwide, with non–small cell lung cancer (NSCLC) accounting for 80%–85% of cases.

HER2 alterations, most commonly exon 20 insertions, occur in 2%–4% of NSCLC cases and are predominantly found in adenocarcinoma, while being rare and poorly characterized in lung squamous cell carcinoma (LSCC).

### Novel insights

ERBB2 (HER2) exon 20 p.G776V may represent a rare, potentially actionable alteration in recurrent lung squamous cell carcinoma (LSCC).

Trastuzumab deruxtecan showed meaningful activity in HER2-mutant LSCC.

Sequential HER2-targeted therapy with trastuzumab deruxtecan and pyrotinib achieved additional disease control.

## Data Availability

The original contributions presented in the study are included in the article/Supplementary Material, further inquiries can be directed to the corresponding authors.
