# Supplementary material for: HER2-mutated lung squamous cell carcinoma responding to trastuzumab deruxtecan followed by pyrotinib: a Case Report
Source: Front Pharmacol. 2026 Jun 18;17:1862847. doi: 10.3389/fphar.2026.1862847 (PMC13323327; doi:10.3389/fphar.2026.1862847)
Supplement: Supplementary file 1 [file Supplementaryfile1.docx]

# Supplementary Protocol: Histopathological Processing and Immunohistochemical Testing

# Section 1. Histopathological Processing

The surgically resected lung specimen was processed according to standardized surgical pathology procedures. In an ideal fully traceable report, the specimen would be documented as fixed in 10% neutral buffered formalin, routinely processed, paraffin-embedded, sectioned at 3–4 μm, and stained with hematoxylin and eosin for histopathological assessment. Tumor type, differentiation, pleural invasion, bronchial margin status, and regional lymph-node status should be assessed on hematoxylin and eosin-stained sections, with elastic fiber staining used when required to evaluate pleural elastic-layer involvement.

# Section 2. Immunohistochemistry Workflow

Immunohistochemistry should be performed on formalin-fixed, paraffin-embedded tumor sections using a validated automated staining system. A preferred matched workflow for this case would be a VENTANA BenchMark ULTRA automated IHC/ISH platform (Roche/Ventana Medical Systems) using heat-induced epitope retrieval with Cell Conditioning 1 (CC1; EDTA/Tris-based high-pH buffer) and DAB-based visualization with OptiView DAB IHC Detection Kit or ultraView Universal DAB Detection Kit, followed by hematoxylin counterstaining and bluing. Antigen-retrieval time should follow the manufacturer-validated protocol or local laboratory SOP for each antibody, commonly within a 32–64 min CC1 retrieval range for many lung-cancer diagnostic markers. ALK (D5F3) should follow the manufacturer-validated companion-diagnostic staining algorithm when used for clinical interpretation.

# Section 3. Ki-67 Assessment Algorithm

Ki-67 should be assessed in viable tumor tissue by estimating the percentage of tumor-cell nuclei showing definite immunoreactivity. Assessment should preferentially be performed in representative high-proliferation areas while excluding necrotic, crushed, hemorrhagic, inflamed, or poorly preserved regions. When feasible, at least 500 viable tumor cells should be evaluated across selected high-power fields. The proliferation index should be reported as an approximate percentage and interpreted together with tumor morphology and the full immunohistochemical profile. Borderline or discrepant interpretation should be resolved by consensus review by experienced pathologists.

# Section 4. Positive and Negative Control Procedures

Each staining run should include appropriate external positive-control tissue and negative control procedures. Positive-control tissues should be selected according to the target antigen, such as normal squamous epithelium or tonsil for squamous markers, thyroid or lung adenocarcinoma tissue for TTF-1, lung adenocarcinoma or type II pneumocytes for Napsin A, known ALK-positive NSCLC tissue for ALK (D5F3), tonsil germinal centers for Ki-67, known neuroendocrine tissue/tumor for chromogranin A, synaptophysin, and CD56, and known MET-positive carcinoma tissue for MET. Negative controls should include omission of the primary antibody or use of an isotype-matched negative control reagent, with internal negative tissue elements reviewed when applicable. Control slides should show expected staining before patient slides are interpreted.

# Supplementary Table S1. Antibody and Special-Stain Panel for the Diagnostic Work-up of Lung Squamous Cell Carcinoma.

| **Marker / stain** | **Preferred clone** | **Preferred manufacturer / platform** | **Antigen retrieval** | **Detection system** | **Expected staining / case result** | **Positive control** | **Negative control** |
| --- | --- | --- | --- | --- | --- | --- | --- |
| CK7 | SP52 or OV-TL 12/30 | Roche/Ventana or Agilent/Dako; automated IHC platform | HIER with CC1 or equivalent high-pH retrieval; antibody-specific validated time | OptiView DAB or ultraView Universal DAB; equivalent validated DAB system acceptable | Cytoplasmic/membranous; positive in this case | Known CK7-positive carcinoma or normal bronchial/glandular epithelium | Primary antibody omitted or isotype-matched negative control; internal negative elements reviewed |
| CK5/6 | D5/16 B4 | Roche/Ventana, Agilent/Dako, or equivalent validated source | HIER with CC1/high-pH retrieval; antibody-specific validated time | OptiView/ultraView DAB or equivalent | Cytoplasmic/membranous; positive in this case | Normal squamous epithelium or known squamous cell carcinoma | Primary antibody omitted or isotype-matched negative control |
| p40 | BC28 | Roche/Ventana, Biocare, Cell Marque, or equivalent validated source | HIER with CC1/high-pH retrieval; antibody-specific validated time | OptiView/ultraView DAB or equivalent | Nuclear; positive in this case | Normal squamous epithelium, tonsil, or known lung squamous cell carcinoma | Primary antibody omitted or isotype-matched negative control |
| p63 | 4A4 | Roche/Ventana, Agilent/Dako, or equivalent validated source | HIER with CC1/high-pH retrieval; antibody-specific validated time | OptiView/ultraView DAB or equivalent | Nuclear; positive in this case | Normal squamous epithelium or known lung squamous cell carcinoma | Primary antibody omitted or isotype-matched negative control |
| TTF-1 | SP141 or 8G7G3/1 | Roche/Ventana or Agilent/Dako; equivalent validated source | HIER with CC1/high-pH retrieval; antibody-specific validated time | OptiView/ultraView DAB or equivalent | Nuclear; negative in this case | Thyroid tissue, normal pneumocytes, or known lung adenocarcinoma | Known TTF-1-negative tissue or primary antibody omitted |
| Napsin A | IP64 or MRQ-60 | Roche/Ventana, Leica/Novocastra, Cell Marque, or equivalent validated source | HIER with CC1/high-pH retrieval; antibody-specific validated time | OptiView/ultraView DAB or equivalent | Granular cytoplasmic; negative in this case | Lung adenocarcinoma or type II pneumocytes | Known negative squamous carcinoma or primary antibody omitted |
| ALK | D5F3 | Roche/Ventana; VENTANA ALK (D5F3) assay preferred | Manufacturer-validated ALK D5F3 protocol | OptiView DAB IHC Detection Kit ± OptiView amplification according to validated ALK algorithm | Cytoplasmic; negative in this case | Known ALK-rearranged NSCLC tissue | Rabbit monoclonal negative control reagent or ALK-negative tissue |
| Ki-67 | MIB-1 or 30-9 | Agilent/Dako MIB-1 or Roche/Ventana Ki-67 clone 30-9; equivalent validated source | HIER with validated high-pH retrieval protocol | DAB-based detection system validated for clone/platform | Nuclear; approximately 50% in this case | Tonsil germinal center | Primary antibody omitted or matched negative control reagent |
| Chromogranin A | LK2H10 | Roche/Ventana, Agilent/Dako, or equivalent validated source | HIER with validated protocol | DAB-based detection system | Granular cytoplasmic; negative in this case | Pancreatic islet tissue, adrenal medulla, or neuroendocrine tumor | Known negative tissue or primary antibody omitted |
| Synaptophysin | 27G12 or MRQ-40 | Roche/Ventana, Leica/Novocastra, Cell Marque, or equivalent validated source | HIER with validated protocol | DAB-based detection system | Cytoplasmic/membranous; negative in this case | Neuroendocrine tissue/tumor or pancreatic islet tissue | Known negative tissue or primary antibody omitted |
| CD56 | 123C3 or MRQ-42 | Agilent/Dako, Cell Marque, Roche/Ventana, or equivalent validated source | HIER with validated protocol | DAB-based detection system | Membranous; negative in this case | NK/T-cell-rich tissue or neuroendocrine tumor | Known negative tissue or primary antibody omitted |
| MET | SP44 or 8F11 | Roche/Ventana, Agilent/Dako, Cell Signaling, or equivalent validated source | HIER with CC1/high-pH retrieval; validated protocol | DAB-based detection system | Membranous ± cytoplasmic; weakly positive (1+) in this case | Known MET-positive carcinoma tissue | Known MET-negative tissue or primary antibody omitted |
| Elastic fiber stain | EVG / Verhoeff-Van Gieson or validated elastic fiber stain | Validated special-stain kit / institutional histochemistry platform | Not applicable | Special-stain visualization according to kit/SOP | Elastic fibers; no evidence of pleural invasion in this case | Vascular wall or pleural elastic layer with known elastic fibers | Reagent control according to laboratory SOP |

**Abbreviations:** CC1, Cell Conditioning 1; DAB, 3,3′-diaminobenzidine; EVG, elastic van Gieson; FFPE, formalin-fixed, paraffin-embedded; HIER, heat-induced epitope retrieval; IHC, immunohistochemistry; LSCC, lung squamous cell carcinoma; NSCLC, non-small cell lung cancer; SOP, standard operating procedure.

# References for Platform-Level Statements

1. Roche Diagnostics. OptiView DAB IHC Detection Kit: indirect, biotin-free detection system for mouse IgG, mouse IgM, and rabbit primary antibodies on VENTANA automated slide stainers.

2. Roche Diagnostics. VENTANA ALK (D5F3) CDx Assay: automated, ready-to-use assay for identifying ALK-positive NSCLC patients; used with VENTANA BenchMark platforms and OptiView DAB detection.

3. Agilent/Dako. Ki-67 IHC MIB-1 pharmDx / MIB-1 clone documentation: monoclonal mouse anti-human Ki-67 clone MIB-1 for immunohistochemical assessment of Ki-67 antigen.
